# Prognostic associations of urine output in AKI and dialysis vintage in ESKD at continuous kidney replacement therapy initiation in ICU patients

**DOI:** 10.1186/s40560-026-00929-9

**Published:** 2026-08-16

**Authors:** Keisuke Okamoto, Naoki Ozu, Hidetada Fukushima, Masahiko Kawaguchi, Kazuhiko Tsuruya

**Affiliations:** 1 Department of Nephrology, Nara Medical University, 840 Shijo-cho, Kashihara, Nara 634-8521 Japan; 2 Institute of Clinical and Translational Science, Nara Medical University, Kashihara, Nara Japan; 3 Department of Emergency and Critical Care Medicine, Nara Medical University, Kashihara, Nara Japan; 4 Department of Anesthesiology, Nara Medical University, Kashihara, Nara Japan

**Keywords:** Acute kidney injury, End-stage kidney disease, Continuous kidney replacement therapy, Urine output, Dialysis vintage, Prognosis

## Abstract

**Background:**

Prognostic assessment at continuous kidney replacement therapy (CKRT) initiation remains challenging in ICU practice. CKRT recipients are heterogeneous; most have acute kidney injury (AKI), whereas a clinically important subset has end-stage kidney disease (ESKD). We evaluated whether pre-CKRT urine output in AKI and dialysis vintage in ESKD were associated with short-term prognosis after CKRT initiation.

**Methods:**

We conducted a single-center retrospective cohort study of consecutive adults who started CKRT in a combined medical–surgical ICU between 2012 and 2021. Patients were classified at CKRT initiation as ESKD, oliguric AKI, or non-oliguric AKI. ESKD was defined as maintenance hemodialysis or peritoneal dialysis before ICU admission. Among patients with AKI, oliguric and non-oliguric AKI were defined as pre-CKRT urine output <0.5 and ≥0.5 mL/kg/h, respectively. The primary outcome was time to all-cause death within 90 days after CKRT initiation. We used Cox proportional hazards models adjusted for prespecified covariates measured at CKRT initiation. Within ESKD, we modeled dialysis vintage as a continuous variable using Cox models adjusted for APACHE II score and sepsis.

**Results:**

Among 560 patients, 66 had ESKD, 305 had oliguric AKI, and 189 had non-oliguric AKI. Documented death within 90 days occurred in 236 patients (42.1%). Using ESKD as the reference, oliguric AKI was not associated with a different hazard of death within 90 days (adjusted hazard ratio [HR], 0.86; 95% confidence interval [CI], 0.58–1.29), whereas non-oliguric AKI was associated with a lower hazard (adjusted HR, 0.63; 95% CI 0.41–0.99). Among patients with ESKD, longer dialysis vintage was associated with a higher hazard of death within 90 days (adjusted HR per 1-year increase, 1.07; 95% CI 1.02–1.11).

**Conclusions:**

In ICU patients starting CKRT, pre-CKRT urine output in AKI and dialysis vintage in ESKD were associated with short-term prognosis. Non-oliguric AKI was associated with a lower adjusted hazard of death than ESKD, and longer dialysis vintage was associated with a higher hazard of death within ESKD. These findings require external validation in contemporary multicenter cohorts.

**Supplementary Information:**

The online version contains supplementary material available at https://doi.org/10.1186/s40560-026-00929-9.

## Introduction

Prognostic assessment at the initiation of continuous kidney replacement therapy (CKRT) remains challenging in ICU practice. At CKRT initiation, clinicians need short-term prognostic information to support family communication and decisions about whether to initiate, continue, or discontinue CKRT [1, 2]. Nonetheless, translating readily available bedside information into reliable short-term prognostic assessment at CKRT initiation remains difficult.

CKRT recipients in the ICU are heterogeneous. Most have acute kidney injury (AKI), whereas a smaller but clinically important subgroup has end-stage kidney disease (ESKD) and receives CKRT during critical illness. ICU teams often manage these populations concurrently, and prognostic assessment may therefore require variables that capture heterogeneity within each condition. In AKI, urine output provides an immediately available measure of severity and predicts mortality independently of overall illness severity [3]. In ESKD, dialysis vintage, defined as the duration of maintenance dialysis before an index time point, is associated with long-term mortality and may reflect cumulative comorbidity burden and reduced physiologic reserve during critical illness [4, 5]. However, urine output and dialysis vintage have not been evaluated together within a unified framework for short-term outcomes among ICU patients receiving CKRT.

To situate the present work, we conducted a predefined search in PubMed and Embase (October 21, 2025) and identified eleven peer-reviewed ICU studies that included comparisons between AKI and ESKD patients receiving acute kidney replacement therapy, including CKRT (details in Additional file 1: Files 1–3). Mortality findings were inconsistent across reports [6–16]. A plausible contributor is limited stratification by urine output at CKRT initiation and the absence of dialysis vintage assessment within ESKD, which may lead to important case-mix differences across cohorts. A recent study reported higher ICU mortality in oliguric than in non-oliguric AKI patients; however, it excluded ESKD patients and was not restricted to CKRT recipients, leaving outcomes under CKRT and comparisons with ESKD uncertain [17].

Our primary objective was to compare adjusted 90-day mortality after CKRT initiation among patients with oliguric AKI, non-oliguric AKI, and ESKD. Our secondary objective was to evaluate the association between dialysis vintage and 90-day mortality within the ESKD group. We additionally performed an exploratory four-group analysis combining AKI urine output categories and ESKD dialysis vintage categories.

## Methods

### Study design and setting

We conducted a single-center retrospective cohort study at Nara Medical University Hospital, a tertiary-care academic center with a combined medical and surgical ICU. We identified consecutive adult patients who received CKRT in the ICU between January 1, 2012 and December 31, 2021. The study protocol was approved by the ethics committee of Nara Medical University Hospital (Approval No. 3288), and the requirement for informed consent was waived owing to the retrospective design.

### Participants

We included patients aged ≥20 years who received CKRT in the ICU for AKI or ESKD during the study period. ESKD was defined as receipt of maintenance hemodialysis or peritoneal dialysis before ICU admission. Patients without pre-existing ESKD were classified as having AKI when CKRT was initiated for acute kidney dysfunction or its complications. We excluded patients who died within 24 h after ICU admission, those who underwent CKRT for non-renal indications, and those who received CKRT for more than 28 days. Non-renal indications were identified from the medical record and referred to CKRT initiated for purposes other than management of AKI or ESKD, including cytokine removal or drug removal.

### Data sources and variables

We abstracted all data from the electronic medical record. AKI and ESKD status and the indication for CKRT initiation were determined from admission notes, ICU progress notes, nephrology consultation notes, dialysis records, and CKRT order records. Patients receiving maintenance hemodialysis or peritoneal dialysis before ICU admission were classified as having ESKD and were not classified as having AKI. Baseline variables at CKRT initiation included age, sex, body mass index (BMI), body weight, mean arterial pressure (MAP), APACHE II score, comorbidities such as hypertension and diabetes mellitus, baseline serum creatinine, cause of AKI, presence of sepsis, main ICU admission diagnosis, mechanical ventilation use, vasopressor requirement, extracorporeal membrane oxygenation (ECMO) use, intra-aortic balloon pumping (IABP) use, CKRT modality, anticoagulation type, initial CKRT settings, and laboratory data. Sepsis was defined by physician-documented sepsis or septic shock in admission, ICU, or nephrology consultation notes. Laboratory variables included hemoglobin, C-reactive protein (CRP), serum albumin, blood urea nitrogen (BUN), serum creatinine, serum potassium, arterial pH, and bicarbonate. We calculated APACHE II within the first 24 h of ICU admission. Pre-CKRT urine output was assessed using available urine output records within 24 h before CKRT initiation, including records from the ICU, ward, or operating room when available. The assessment window was capped at 24 h. Urine output was standardized by body weight and the assessment window and expressed as mL/kg/h. We also recorded the interval from ICU admission to CKRT initiation and the duration of the pre-CKRT urine output assessment window. The main ICU admission diagnosis was classified according to the primary reason for ICU admission. The original diagnosis categories were grouped into seven categories: sepsis or septic shock, cardiovascular disease, other shock, respiratory failure, cardiopulmonary arrest, postoperative or surgical status, and other diagnoses. Cardiovascular disease included cardiogenic shock, acute decompensated heart failure without shock, pericardial effusion or tamponade, and acute myocardial infarction. We recorded initial CKRT settings at CKRT initiation, including blood flow rate, dialysate flow rate, replacement fluid flow rate, CKRT modality, anticoagulation type, and hemofilter membrane type. Initial prescribed CKRT dose was calculated as the sum of the initial dialysate flow rate and replacement fluid flow rate divided by body weight. As a crude marker of cardiopulmonary congestion at CKRT initiation, we recorded chest radiography findings. Radiographic congestion was defined as the presence of any of the following: an enlarged cardiothoracic ratio suggestive of cardiomegaly, pulmonary congestion, or pleural effusion (yes/no).

### Exposures and outcome

*Main exposure* The main exposure was a three-group classification at CKRT initiation: ESKD, oliguric AKI, and non-oliguric AKI. ESKD was defined as receipt of maintenance hemodialysis or peritoneal dialysis before ICU admission. Among patients with AKI, oliguric AKI was defined as pre-CKRT urine output <0.5 mL/kg/h, and non-oliguric AKI was defined as pre-CKRT urine output ≥0.5 mL/kg/h.

*Secondary exposure* The secondary exposure was dialysis vintage in the ESKD group. Dialysis vintage was defined as the duration of maintenance dialysis before CKRT initiation and was modeled primarily as a continuous variable in years.

*Outcome* The primary outcome was time to all-cause death within 90 days after CKRT initiation. Time zero was defined as CKRT initiation. Mortality was ascertained from the electronic medical record of our hospital. Patients without documented death within 90 days were followed until the last date with available hospital records or 90 days after CKRT initiation, whichever came first. Patients who had no documented death and whose available follow-up ended before 90 days were treated as right censored at the last documented follow-up time.

### Statistical analysis

We summarized continuous variables as medians (interquartile ranges) and categorical variables as counts (percentages). We compared baseline characteristics across groups using the Kruskal–Wallis test for continuous variables (and the Mann–Whitney U test for two-group comparisons) and Pearson’s χ^2^ test for categorical variables.

*Main exposure analysis (three-group comparison)* We used univariable and multivariable Cox proportional hazards models to estimate unadjusted and adjusted hazard ratios (HRs) with 95% confidence intervals (CIs) for time to all-cause death within 90 days after CKRT initiation across the three groups, using the ESKD group as the reference. We plotted survival curves using the Kaplan–Meier method and compared groups using the log-rank test. For the multivariable Cox model, we adjusted for covariates measured at CKRT initiation, which were prespecified based on clinical relevance and prior literature, including age, sex, MAP, BMI, APACHE II score, diabetes mellitus, sepsis, mechanical ventilation use, vasopressor use, hemoglobin, serum albumin, and CRP [3, 17–27].

*Secondary analysis (within ESKD)* In the ESKD group, we fitted univariable and multivariable Cox proportional hazards models with dialysis vintage (years) as a continuous variable. We modeled the adjusted association between dialysis vintage and time to all-cause death within 90 days after CKRT initiation using restricted cubic splines with 3 knots placed at the 10th, 50th, and 90th percentiles of dialysis vintage, corresponding to 1.11, 5.58, and 19.92 years, and reported P values for the overall association and for non-linearity. Given the limited number of events in the ESKD group, we used a parsimonious adjustment set including APACHE II score and sepsis.

*Sensitivity and exploratory analyses* For the main exposure analysis, we performed four sensitivity analyses. First, we additionally adjusted the primary Cox model for the initial prescribed CKRT dose, which was available at CKRT initiation [28]. Second, we further adjusted the primary Cox model for radiographic congestion at CKRT initiation. Third, we repeated the primary Cox model after restricting the AKI cohort to patients with a pre-CKRT urine output assessment window of at least 6 h, while retaining all ESKD patients. Fourth, we performed a two-interval landmark Cox regression analysis for 0–7 days and 7–90 days after CKRT initiation. The landmark Cox models used the same baseline covariate set as the primary Cox model. As an exploratory analysis, we dichotomized the ESKD cohort at the median dialysis vintage and evaluated a four-group classification: ESKD long-vintage, ESKD short-vintage, oliguric AKI, and non-oliguric AKI. ESKD long-vintage was used as the reference group. This analysis was considered exploratory because the median dialysis vintage was not intended to represent a clinically established threshold. Kaplan–Meier curves and univariable and multivariable Cox regression results for this exploratory four-group analysis are presented in the Supplementary material. We handled missing data using complete-case analysis; serum albumin and radiographic congestion had 0.5% missingness. All tests were two sided, and we considered P < 0.05 statistically significant. Analyses were performed using R version 4.4.0 (R Foundation for Statistical Computing). All authors had full access to all data in the study and had final responsibility for the decision to submit for publication.

## Results

### Patient flow

**Fig. 1:**
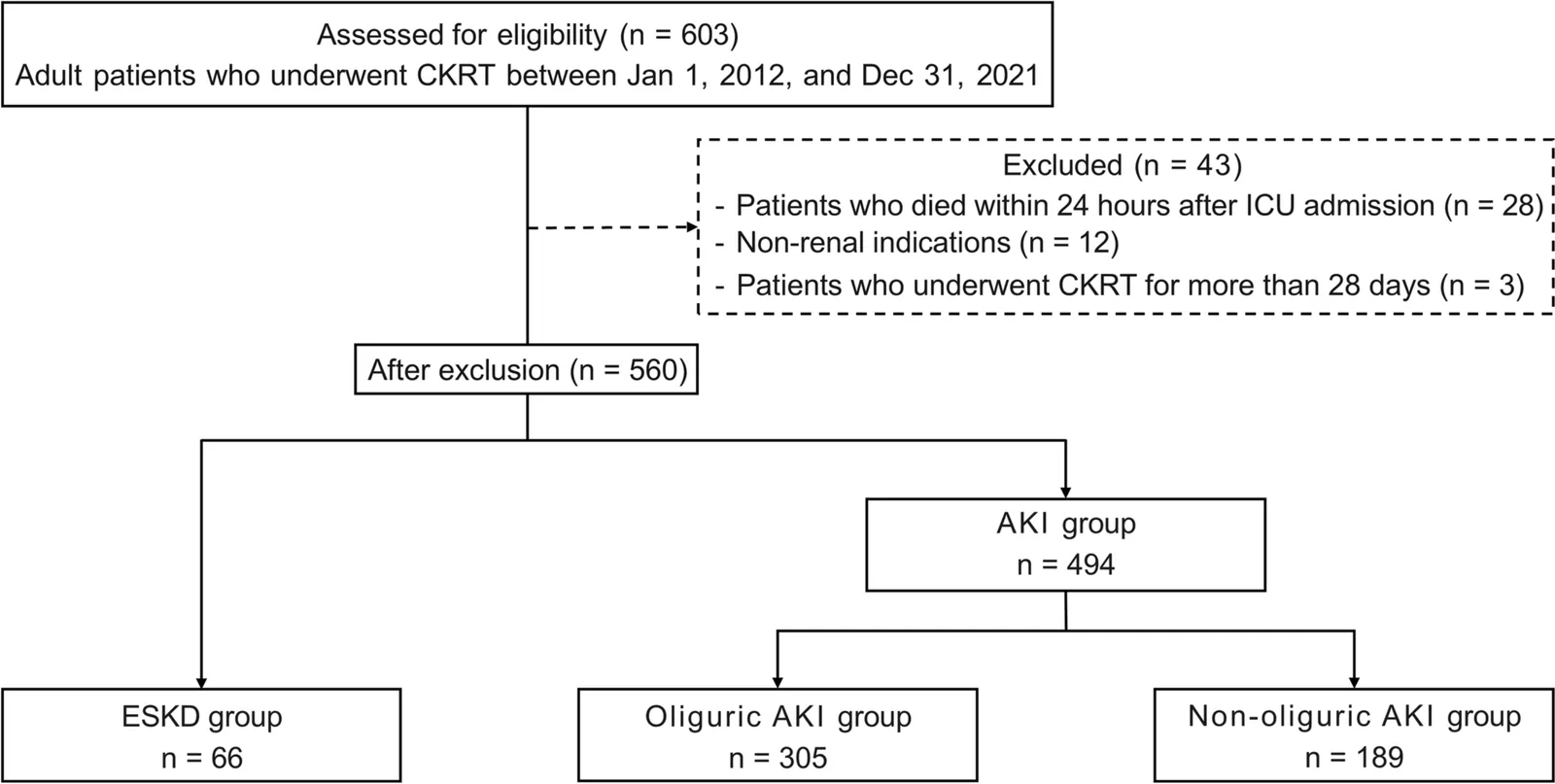
Patient flow chart. *AKI* acute kidney injury, *CKRT* continuous kidney replacement therapy, *ESKD* end-stage kidney disease, *ICU* intensive care unit. Oliguric AKI was defined as pre-CKRT urine output <0.5 mL/kg/h, and non-oliguric AKI as pre-CKRT urine output ≥0.5 mL/kg/h. ESKD indicates patients receiving maintenance hemodialysis or peritoneal dialysis

Among 603 patients assessed for eligibility, 560 met the inclusion criteria and were analyzed (Fig. 1). The cohort included 66 ESKD patients and 494 AKI patients. Based on pre-CKRT urine output standardized by body weight and assessment window, the AKI group was classified as oliguric AKI (*n* = 305) or non-oliguric AKI (*n* = 189) at CKRT initiation.

### Baseline patient and CKRT characteristics

Baseline patient characteristics are shown in Table 1. This study included 66 patients in the ESKD group, 305 in the oliguric AKI group, and 189 in the non-oliguric AKI group. Median age was 71 years in the ESKD group, 74 years in the oliguric AKI group, and 70 years in the non-oliguric AKI group. Median APACHE II scores were 26, 26, and 23, respectively. Diabetes mellitus was more common in the ESKD group (50.0%) than in the oliguric AKI group (28.9%) and the non-oliguric AKI group (24.9%). ICU admission diagnosis also differed across groups. In contrast, sex, BMI, MAP, sepsis, mechanical ventilation, vasopressor use, ECMO use, IABP use, and CRP were similar across groups. Hemoglobin and serum albumin differed across groups. Radiographic congestion at CKRT initiation was highly prevalent and similar across groups.

**Table 1:** Baseline patient characteristics

Characteristics	Overall (*n* = 560)	ESKD (*n* = 66)	Oliguric AKI (*n* = 305)	Non-oliguric AKI (*n* = 189)	*P*
Age, years	72 [62, 80]	71 [64, 77]	74 [64, 81]	70 [58, 80]	0.094
Sex (Male), *n* (%)	356 (63.6)	47 (71.2)	198 (64.9)	111 (58.7)	0.148
Body mass index, kg/m^2^	22.2 [19.5, 25.2]	22.7 [19.0, 25.0]	22.2 [19.8, 25.9]	22.1 [19.1, 24.6]	0.266
Body weight, kg	56.5 [48.0, 67.0]	56.1 [47.7, 67.5]	56.2 [48.0, 67.4]	56.7 [46.5, 64.8]	0.629
Hypertension, *n* (%)	294 (52.5)	41 (62.1)	165 (54.1)	88 (46.6)	0.066
Diabetes mellitus, *n* (%)	168 (30.0)	33 (50.0)	88 (28.9)	47 (24.9)	0.001
Baseline serum creatinine, mg/dL	0.9 [0.7, 1.3]	NA	0.9 [0.7, 1.3]	0.8 [0.6, 1.1]	NA
Mean arterial pressure, mmHg	76 [63, 89]	76 [62, 82]	74 [63, 89]	79 [65, 91]	0.111
APACHE II score	25 [19, 30]	26 [22, 32]	26 [20, 31]	23 [18, 29]	0.001
Sepsis, *n* (%)	263 (47.0)	30 (45.5)	150 (49.2)	83 (43.9)	0.505
Mechanical ventilation, *n* (%)	391 (69.8)	44 (66.7)	213 (69.8)	134 (70.9)	0.812
Vasopressor use, *n* (%)	404 (72.1)	46 (69.7)	225 (73.8)	133 (70.4)	0.64
ECMO use, *n* (%)	29 (5.2)	1 (1.5)	17 (5.6)	11 (5.8)	0.357
IABP use, *n* (%)	4 (0.7)	0 (0.0)	2 (0.7)	2 (1.1)	0.669
Radiographic congestion at CKRT initiation, *n* (%)	554 (99.5)	65 (98.5)	303 (99.7)	186 (99.5)	0.491
Hemoglobin, g/dL	10.0 [8.6, 12.1]	9.7 [8.2, 11.4]	9.9 [8.5, 12.0]	10.4 [9.0, 12.3]	0.032
Serum albumin, g/dL	2.7 [2.3, 3.2]	2.9 [2.4, 3.3]	2.7 [2.3, 3.1]	2.8 [2.3, 3.3]	0.022
Blood urea nitrogen, mg/dL	50 [31, 73]	52 [42, 75]	51 [31, 74]	47 [28, 71]	0.174
Serum creatinine, mg/dL	2.8 [1.8, 4.5]	7.2 [5.5, 8.8]	2.8 [1.9, 4.0]	2.2 [1.4, 3.4]	<0.001
Serum potassium, mEq/L	4.4 [3.8, 5.2]	4.4 [4.0, 5.5]	4.5 [3.8, 5.3]	4.4 [3.7, 5.1]	0.481
C-reactive protein, mg/dL	10.3 [2.7, 19.7]	11.8 [2.8, 18.3]	10.7 [3.9, 19.7]	8.5 [1.4, 19.8]	0.233
pH on ABG	7.35 [7.27, 7.41]	7.36 [7.28, 7.41]	7.34 [7.26, 7.40]	7.36 [7.29, 7.42]	0.037
Bicarbonate on ABG, mEq/L	19.4 [15.8, 22.7]	21.0 [18.1, 23.3]	18.7 [15.1, 21.9]	19.6 [16.1, 23.3]	0.004
ICU admission diagnosis, *n* (%)					0.010
Sepsis/septic shock	263 (47.0)	30 (45.5)	150 (49.2)	83 (43.9)	
Cardiovascular	15 (2.7)	3 (4.5)	5 (1.6)	7 (3.7)	
Respiratory failure	58 (10.4)	7 (10.6)	32 (10.5)	19 (10.1)	
Cardiopulmonary arrest	51 (9.1)	12 (18.2)	22 (7.2)	17 (9.0)	
Postoperative/surgical	19 (3.4)	5 (7.6)	10 (3.3)	4 (2.1)	
Other shock	63 (11.2)	8 (12.1)	35 (11.5)	20 (10.6)	
Other	91 (16.2)	1 (1.5)	51 (16.7)	39 (20.6)	
ICU admission to CKRT initiation, hours	5.0 [2.3, 20.0]	3.7 [1.9, 12.3]	5.3 [2.3, 22.0]	5.0 [2.4, 21.4]	0.108
Pre-CKRT urine output assessment window, hours	9.1 [3.2, 24.0]	NA	10.4 [3.5, 24.0]	7.8 [3.0, 24.0]	0.311
Pre-CKRT urine output, mL/kg/h	0.34 [0.09, 0.77]	NA	0.14 [0.03, 0.29]	1.01 [0.70, 1.66]	<0.001
Cause of AKI, *n* (%)					NA
Acute tubular injury	404 (81.8)	NA	259 (84.9)	145 (76.7)	
Nephrotoxic agent	29 (5.9)	NA	16 (5.2)	13 (6.9)	
Cardiorenal	9 (1.8)	NA	4 (1.3)	5 (2.6)	
Hepatorenal	10 (2.0)	NA	6 (2.0)	4 (2.1)	
Other	15 (3.0)	NA	7 (2.3)	8 (4.2)	
Unknown	27 (5.5)	NA	13 (4.3)	14 (7.4)	

Data are presented as median [interquartile range] or *n* (%). *P* values were calculated across the three groups: ESKD, oliguric AKI, and non-oliguric AKI. For variables assessed only among patients with AKI, P values were calculated between the oliguric AKI and non-oliguric AKI groups. Percentages were calculated using non-missing denominators. Oliguric AKI was defined as pre-CKRT urine output <0.5 mL/kg/h, and non-oliguric AKI was defined as pre-CKRT urine output ≥0.5 mL/kg/h. Pre-CKRT urine output was assessed using available urine output records within 24 h before CKRT initiation, including records from the ICU, ward, or operating room when available. The assessment window was capped at 24 h, and urine output was standardized by body weight and assessment window. Cause of AKI and pre-CKRT urine output variables were assessed only among patients with AKI and were not applicable for patients with ESKD. The main ICU admission diagnosis was classified according to the primary reason for ICU admission. Radiographic congestion at CKRT initiation was defined as the presence of any of the following on chest radiography: an enlarged cardiothoracic ratio suggestive of cardiomegaly, pulmonary congestion, or pleural effusion. Missing data were minimal for variables included in the primary multivariable model

*ABG* arterial blood gas, *AKI* acute kidney injury, *APACHE* Acute Physiology and Chronic Health Evaluation, *CKRT* continuous kidney replacement therapy, *ECMO* extracorporeal membrane oxygenation, *ESKD* end-stage kidney disease, *IABP* intra-aortic balloon pumping, *ICU* intensive care unit, *NA* not applicable

CKRT settings at initiation are shown in Table 2. Most patients received continuous venovenous hemodiafiltration (CVVHDF), and the remaining patients received continuous venovenous hemodialysis (CVVHD). Anticoagulation type and hemofilter membrane type were similar across groups, and nafamostat mesylate was used in approximately 90% of patients in each group. Initial blood flow rate, dialysate flow rate, replacement fluid flow rate, and initial prescribed CKRT dose were also similar across groups.

**Table 2:** CKRT settings at initiation

Characteristics	Overall (*n* = 560)	ESKD (*n* = 66)	Oliguric AKI (*n* = 305)	Non-oliguric AKI (*n* = 189)	*P*
CKRT modality, *n* (%)					0.460
CVVHDF	556 (99.3)	65 (98.5)	304 (99.7)	187 (98.9)	
CVVHD	4 (0.7)	1 (1.5)	1 (0.3)	2 (1.1)	
Anticoagulation, *n* (%)					0.326
Nafamostat mesylate	502 (89.6)	58 (87.9)	277 (90.8)	167 (88.4)	
Heparin	25 (4.5)	5 (7.6)	13 (4.3)	7 (3.7)	
Heparin + nafamostat mesylate	17 (3.0)	0 (0.0)	9 (3.0)	8 (4.2)	
Argatroban hydrate	2 (0.4)	1 (1.5)	0 (0.0)	1 (0.5)	
None	14 (2.5)	2 (3.0)	6 (2.0)	6 (3.2)	
Hemofilter membrane type, *n* (%)					0.303
AN69ST	121 (21.6)	15 (22.7)	65 (21.3)	41 (21.7)	
PS	435 (77.7)	50 (75.8)	239 (78.4)	146 (77.2)	
CTA	2 (0.4)	0 (0.0)	0 (0.0)	2 (1.1)	
Unknown	2 (0.4)	1 (1.5)	1 (0.3)	0 (0.0)	
Initial blood flow rate, mL/min	100 [80, 150]	100 [100, 150]	100 [80, 150]	100 [100, 150]	0.063
Initial dialysate flow rate, mL/h	400 [100, 500]	300 [100, 500]	400 [100, 500]	400 [100, 500]	0.734
Initial replacement fluid flow rate, mL/h	400 [300, 700]	500 [300, 700]	300 [300, 650]	400 [300, 700]	0.222
Initial prescribed CKRT dose, mL/kg/h	13.9 [11.8, 16.9]	13.7 [11.9, 18.0]	13.8 [11.7, 16.8]	14.0 [11.9, 16.9]	0.388

Data are presented as median [interquartile range] or *n* (%). *P* values were calculated across the three groups: ESKD, oliguric AKI, and non-oliguric AKI. Oliguric AKI was defined as pre-CKRT urine output <0.5 mL/kg/h, and non-oliguric AKI was defined as pre-CKRT urine output ≥0.5 mL/kg/h. CKRT settings, including modality and hemofilter membrane type, indicate initial settings at CKRT initiation. Unknown indicates that hemofilter membrane type was not documented in the CKRT prescription record. Initial prescribed CKRT dose was calculated as the sum of the initial dialysate flow rate and replacement fluid flow rate divided by body weight

*AKI* acute kidney injury, *AN69ST* surface-treated AN69 membrane, *CKRT* continuous kidney replacement therapy, *CTA* cellulose triacetate, *CVVHD* continuous venovenous hemodialysis, *CVVHDF* continuous venovenous hemodiafiltration, *ESKD* end-stage kidney disease, *PS* polysulfone

### Primary outcome

Within 90 days after CKRT initiation, documented death occurred in 236 of 560 patients (42.1%) in the overall cohort: 32 of 66 (48.5%) in the ESKD group, 143 of 305 (46.9%) in the oliguric AKI group, and 61 of 189 (32.3%) in the non-oliguric AKI group. A total of 104 patients (18.6%) had no documented death and had available follow-up ending before 90 days; these patients were treated as right censored at the last documented follow-up time. The number of patients censored before 90 days was 5 of 66 (7.6%) in the ESKD group, 52 of 305 (17.0%) in the oliguric AKI group, and 47 of 189 (24.9%) in the non-oliguric AKI group.

**Fig. 2:**
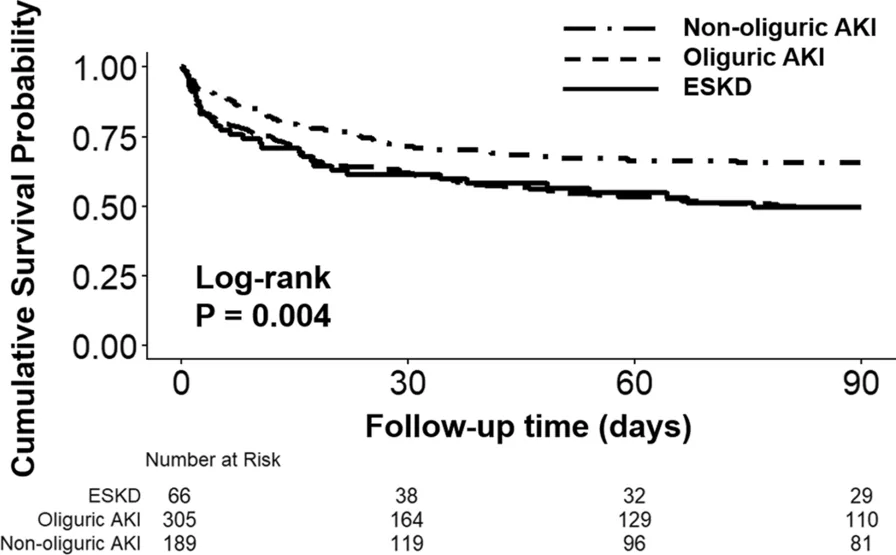
Kaplan–Meier curves for the primary three-group comparison. Kaplan–Meier curves show cumulative survival probability within 90 days after CKRT initiation in the ESKD, oliguric AKI, and non-oliguric AKI groups. Groups were compared using the log-rank test (*P* = 0.004). Numbers at risk are shown below the plot. Oliguric AKI was defined as pre-CKRT urine output <0.5 mL/kg/h, and non-oliguric AKI was defined as pre-CKRT urine output ≥0.5 mL/kg/h. *AKI* acute kidney injury, *CKRT* continuous kidney replacement therapy, *ESKD* end-stage kidney disease

Kaplan–Meier curves for the primary three-group comparison are shown in Fig. 2. Survival differed across the three groups (log-rank test, P = 0.004). In the univariable Cox regression analysis using the ESKD group as the reference, oliguric AKI was not associated with a different hazard of death within 90 days (HR, 0.99; 95% CI 0.68–1.46; *P* = 0.974), whereas non-oliguric AKI was associated with a lower hazard of death within 90 days (HR, 0.61; 95% CI 0.40–0.94; *P* = 0.025). In the multivariable Cox regression analysis adjusted for prespecified covariates measured at CKRT initiation, the adjusted HR was 0.86 (95% CI 0.58–1.29; *P* = 0.475) for oliguric AKI and 0.63 (95% CI 0.41–0.99; *P* = 0.044) for non-oliguric AKI (Table 3).

**Table 3:** Univariable and multivariable Cox regression analyses for 90-day mortality

Group	Deaths/*N* (%)	Unadjusted HR (95% CI)	*P*	Adjusted HR (95% CI)	*P*
ESKD	32/66 (48.5)	Reference		Reference	
Oliguric AKI	143/305 (46.9)	0.99 (0.68–1.46)	0.974	0.86 (0.58–1.29)	0.475
Non-oliguric AKI	61/189 (32.3)	0.61 (0.40–0.94)	0.025	0.63 (0.41–0.99)	0.044

Hazard ratios were estimated using Cox proportional hazards models with ESKD as the reference group. The multivariable model was adjusted for age, sex, mean arterial pressure, body mass index, APACHE II score, diabetes mellitus, sepsis, mechanical ventilation use, vasopressor use, hemoglobin, serum albumin, and C-reactive protein. Oliguric AKI was defined as pre-CKRT urine output <0.5 mL/kg/h, and non-oliguric AKI was defined as pre-CKRT urine output ≥0.5 mL/kg/h

*AKI* acute kidney injury, *APACHE* Acute Physiology and Chronic Health Evaluation, *CI* confidence interval, *CKRT* continuous kidney replacement therapy, *ESKD* end-stage kidney disease, *HR* hazard ratio

### Secondary analysis (within ESKD)

**Fig. 3:**
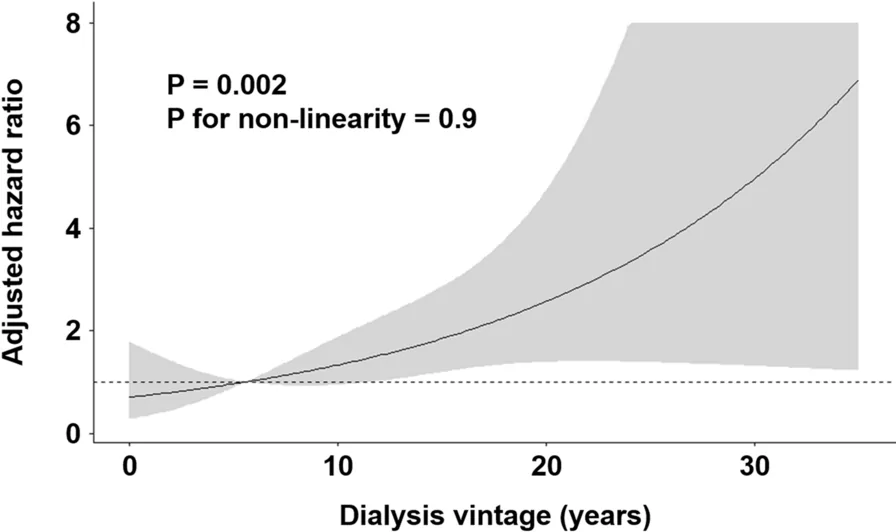
Association between dialysis vintage and 90-day mortality in patients with ESKD receiving CKRT. The solid line shows adjusted hazard ratios (HRs) estimated using a restricted cubic spline for dialysis vintage (years), and the shaded area indicates 95% confidence intervals. The reference was the median dialysis vintage (5.6 years; HR = 1.0). The Cox model was adjusted for APACHE II score and sepsis. P values correspond to the overall association (*P* = 0.002) and the test for non-linearity (*P* = 0.9). *CKRT* continuous kidney replacement therapy, *ESKD* end-stage kidney disease

Among patients in the ESKD group, we evaluated the association between dialysis vintage and time to all-cause death within 90 days after CKRT initiation. The ESKD group included 66 patients, of whom 32 (48.5%) died within 90 days. Median dialysis vintage was 5.6 years (IQR, 2.6–11.3). In the multivariable Cox model adjusted for APACHE II score and sepsis, longer dialysis vintage, modeled as a continuous variable, was associated with a higher hazard of death within 90 days (adjusted HR per 1-year increase, 1.07; 95% CI 1.02–1.11; *P* = 0.002). The adjusted association modeled with a restricted cubic spline is shown in Fig. 3, with the median dialysis vintage (5.6 years) as the reference (HR = 1.0). There was no evidence of non-linearity (*P* for non-linearity = 0.9).

### Sensitivity analyses

We performed four sensitivity analyses to evaluate the robustness of the primary findings. First, after additional adjustment for the initial prescribed CKRT dose, the estimates were similar to those in the primary model. Using the ESKD group as the reference, the adjusted HR for death within 90 days was 0.86 (95% CI 0.58–1.29; *P* = 0.478) for oliguric AKI and 0.63 (95% CI 0.41–0.99; *P* = 0.045) for non-oliguric AKI.

Second, after additional adjustment for radiographic congestion at CKRT initiation, the estimates were also similar to those in the primary model. Using the ESKD group as the reference, the adjusted HR for death within 90 days was 0.87 (95% CI 0.58–1.30; *P* = 0.490) for oliguric AKI and 0.64 (95% CI 0.41–0.99; *P* = 0.046) for non-oliguric AKI.

Third, after restricting the AKI cohort to patients with a pre-CKRT urine output assessment window of at least 6 h while retaining all ESKD patients, 360 patients were included. The adjusted HRs for death within 90 days were 1.08 (95% CI 0.70–1.65; *P* = 0.730) for oliguric AKI and 0.76 (95% CI 0.46–1.25; *P* = 0.286) for non-oliguric AKI. The estimate for non-oliguric AKI was imprecise and not statistically significant.

Fourth, in the two-interval landmark Cox analysis, the adjusted HRs for death during 0–7 days were 0.80 (95% CI 0.45–1.42; *P* = 0.441) for oliguric AKI and 0.56 (95% CI 0.29–1.08; *P* = 0.081) for non-oliguric AKI, using ESKD as the reference. During 7–90 days, the adjusted HRs were 0.93 (95% CI 0.52–1.64; *P* = 0.794) for oliguric AKI and 0.71 (95% CI 0.39–1.31; *P* = 0.277) for non-oliguric AKI (Additional file 1: File 4). The point estimates for non-oliguric AKI were below 1.0 in both intervals, but the estimates were imprecise.

### Exploratory analysis

We performed an exploratory four-group analysis that stratified the ESKD group by the median dialysis vintage in the ESKD cohort (5.6 years). Baseline patient characteristics and CKRT settings among ESKD patients stratified by dialysis vintage are shown in Additional file 1: Files 5 and 6. The group sizes and numbers of deaths within 90 days were 33 patients and 20 deaths (60.6%) in the ESKD long-vintage group, 33 patients and 12 deaths (36.4%) in the ESKD short-vintage group, 305 patients and 143 deaths (46.9%) in the oliguric AKI group, and 189 patients and 61 deaths (32.3%) in the non-oliguric AKI group. Kaplan–Meier curves for this exploratory analysis are shown in Additional file 1: File 7. Using ESKD long-vintage as the reference, the adjusted HR for death within 90 days was 0.41 (95% CI 0.20–0.85; *P* = 0.017) for ESKD short-vintage, 0.57 (95% CI 0.35–0.92; *P* = 0.022) for oliguric AKI, and 0.42 (95% CI 0.25–0.70; *P* = 0.001) for non-oliguric AKI (Additional file 1: File 8). This analysis was considered exploratory because dialysis vintage was dichotomized at the median value rather than at a clinically established threshold, and the ESKD subgroups were small.

## Discussion

In this single-center retrospective cohort of ICU patients who started CKRT, pre-CKRT urine output in patients with AKI and dialysis vintage in patients with ESKD were associated with time to all-cause death within 90 days after CKRT initiation. Oliguric AKI was not associated with a different adjusted hazard of death compared with ESKD, whereas non-oliguric AKI was associated with a lower adjusted hazard. Sensitivity analyses adjusting for initial prescribed CKRT dose or radiographic congestion showed similar estimates, whereas analyses restricted to longer urine output assessment windows were less precise. The landmark analysis should be interpreted cautiously because its estimates were imprecise and may depend on the selected 7-day boundary; therefore, it does not exclude prognostic separation over a longer early period, such as 14 days. The early separation of survival curves may reflect differences in residual urine output, fluid tolerance, and cardiovascular vulnerability at CKRT initiation, although these mechanisms remain speculative. Within the small ESKD cohort, longer dialysis vintage, modeled as a continuous variable, was associated with a higher hazard of death within 90 days. These findings suggest that routinely available information at CKRT initiation may help describe prognostic heterogeneity among patients requiring CKRT, although this study did not develop or validate a formal prediction model.

Table 4 summarizes prior observational studies that compared outcomes between AKI and ESKD in critically ill patients treated with KRT, including CKRT, and reported inconsistent mortality findings across varying outcome definitions and follow-up windows [6–16]. These eleven studies were identified through a predefined structured search, with details provided in Additional file 1: Files 1–3. Beyond heterogeneity in AKI definitions and KRT modality reporting, Table 4 indicates limited reporting of urine output within AKI and dialysis vintage within ESKD. Urine output handling, as defined in Table 4, was not reported in most studies (9 of 11), and only two studies reported urine output descriptively without incorporating urine output into the primary analysis. Dialysis vintage was not reported in any study. Because these factors were seldom reported, the existing literature cannot determine whether differences in pre-CKRT urine output among AKI patients or dialysis vintage among ESKD patients contributed to inconsistent mortality findings across studies. We therefore view urine output and dialysis vintage as hypothesis-generating contributors to between-study heterogeneity.

**Table 4:** Prior observational studies comparing outcomes between AKI and ESKD in critically ill patients receiving kidney replacement therapy

Study (first author, year)	Study period (years)	Design	Country	KRT modality	Outcome (time point)	Group sizes (deaths, %)		Main conclusion	Urine output handling	Dialysis vintage handling	AKI definition
						AKI	ESKD				
Ostermann (2008) [6]	1989–1999	Retro, MC	UK; Germany	NR	ICU mortality	1,847; deaths ≈999 (54.1%)	797; deaths ≈166 (20.8%)	AKI worse	NR	NR	KRT- or creatinine-based^d^
Clermont (2002) [7]	10 mo (years NR)	Retro, SC	USA	Mixed	ICU mortality	254; deaths=59 (23%)	57; deaths=6 (11%)	AKI worse	Reported only	NR	Creatinine-based^b^
Uchino (2003) [8]	3 mo (years NR)	Pros, MC	Australia	CKRT only	ICU mortality	32; deaths=12 (37.5%)	32; deaths=7 (21.9%)	No difference (NS)	NR	NR	Creatinine-based^e^
Akbaş (2015) [9]	2000–2007	Retro, SC	Turkey	Mixed	ICU mortality	115; deaths=78 (67.8%)	101; deaths=51 (50.5%)	AKI worse	NR	NR	KRT-based^a^
Walcher (2011) [10]	2002–2010	Retro, SC	USA	CKRT only	ICU mortality	108; deaths=66 (61%)	28; deaths=10 (36%)	AKI worse	NR	NR	KRT-based^f^
Hussain (2009) [11]	2003–2005	Retro, SC	USA	CKRT only	In-hospital mortality	86; deaths=54 (63%)	24; deaths=11 (46%)	AKI worse	NR	NR	KRT-based^g^
Rocha (2009) [12]	2004–2007	Pros, MC	Brazil	Mixed	ICU mortality	54; deaths=23 (43%)	54; deaths=11 (20%)	AKI worse	Reported only	NR	KRT-based^h^
Lohse (2019) [13]	2005–2012	Retro, SC	Denmark	NR	90-day mortality	1,004; deaths≈552 (55%)	161; deaths≈72 (45%)	No difference (NS)	NR	NR	KRT-based^a^
Kao (2017) [14]	2007–2011	Retro, Natl DB	Taiwan	CKRT only	In-hospital mortality	13,204; deaths=8844 (67.0%)	2,249; deaths=1423 (63.3%)	No difference (NS)	NR	NR	Administrative codes^c^
Allegretti (2013) [15]	2008–2011	Pros, SC	USA	CKRT only	In-hospital mortality	725; deaths≈440 (60.7%)	138; deaths≈75 (54.3%)	No difference (NS)	NR	NR	KRT-based^a^
Jung (2012) [16]	2009–2010	Retro, SC	Korea	CKRT only	90-day mortality	76; deaths=43 (56.6%)	24; deaths=10 (41.7%)	No difference (NS)	NR	NR	KRT-based^a^

KRT modality: CKRT only indicates that only continuous modalities were included. Mixed indicates inclusion of intermittent KRT (e.g., IHD, SLED, and/or PD) in addition to CKRT. NR indicates that the KRT modality was not specified. Outcome: Time point reflects the primary short-term outcome reported for the AKI vs. ESKD comparison in each study (ICU mortality, in-hospital mortality, or 90-day mortality, as applicable). Group sizes (deaths, %): When studies reported mortality only as a percentage, the number of deaths was calculated from the percentage and group size and is shown as an approximation (≈); minor discrepancies may occur due to rounding. Main conclusion: “No difference (NS)” indicates that the study reported no statistically significant difference between AKI and ESKD in the primary or adjusted analysis. Urine output handling and dialysis vintage handling: “Reported only” indicates the variable was presented as baseline data but was not used for stratification/subgrouping or multivariable adjustment. “NR” indicates not reported

*AKI* acute kidney injury, *ESKD* end-stage kidney disease, *KRT* kidney replacement therapy, *CKRT* continuous kidney replacement therapy, *ICU* intensive care unit, *SC* single-center, *MC* multicenter, *Retro* retrospective, *Pros* prospective, *Natl DB* national database, *HD* hemodialysis, *PD* peritoneal dialysis, *IHD* intermittent hemodialysis, *SLED* sustained low-efficiency dialysis, *RIFLE* Risk, Injury, Failure, Loss, End-stage kidney disease, *AKIN* Acute Kidney Injury Network, *KDIGO* Kidney Disease: Improving Global Outcomes, *NR* not reported, *NS* not significant

AKI definition:

^a^ Acute KRT initiated during ICU admission (explicit KDIGO/AKIN/RIFLE criteria not specified)

^b^ Hou criteria: ΔCr thresholds by baseline creatinine; ΔCr defined as maximum minus minimum creatinine during ICU stay; patients dialyzed on clinical grounds could be included

^c^ Identified using administrative diagnosis/procedure codes in a national claims database

^d^ AKI defined as KRT requirement and/or serum creatinine rise >300% from baseline

^e^ AKI defined as normal premorbid creatinine (ESKD defined as chronic dialysis [HD/PD])

^f^ CKRT recipients without underlying CKD/ESKD; AKI severity corresponds to AKIN stage 3 or RIFLE-F by design

^g^ Severe AKI requiring KRT with creatinine rise from baseline and/or oliguria/anuria with indications (e.g., fluid overload, acidosis, hyperkalemia)

^h^ AKI requiring KRT within 48 h of ICU stay; severity classified by RIFLE at KRT initiation

Pre-CKRT urine output is a bedside marker that may reflect both the severity and the trajectory of acute kidney dysfunction beyond creatinine-based AKI definitions. In our cohort, the association between non-oliguric AKI and a lower adjusted hazard of death within 90 days persisted after multivariable adjustment, suggesting that urine output may be a clinically informative prognostic marker at the time CKRT is initiated. This direction is consistent with prior ICU cohorts of AKI not restricted to CKRT, in which non-oliguric AKI was associated with better outcomes than oliguric AKI [17, 29]. However, urine output may also act as a proxy for hemodynamic instability, and residual confounding may remain despite adjustment for vasopressor requirement.

Dialysis vintage represents an ESKD-specific source of heterogeneity that is not captured when ESKD is treated as a single reference category. Longer dialysis vintage may reflect cumulative vulnerability, including gradual nutritional decline and increased susceptibility to infection [4, 5]. In our ESKD-restricted analysis, longer dialysis vintage was associated with a higher hazard of death within 90 days, with no evidence of non-linearity in the restricted cubic spline analysis. Because the ESKD cohort was small, this finding should be interpreted cautiously. In the exploratory four-group analysis, differences in pre-CKRT urine output among AKI patients and dialysis vintage among ESKD patients may have contributed to the observed pattern of mortality across groups.

Study period may also help explain variability in mortality findings across prior AKI–ESKD studies of acute KRT. Among the studies summarized in Table 4, earlier cohorts tended to conclude that AKI had higher mortality than ESKD, whereas more contemporary cohorts tended to report no statistically significant difference. This apparent time trend does not establish causality and may reflect changes in ICU case mix, thresholds for starting KRT, and critical care practice. Long-term survival among maintenance hemodialysis patients has improved, and long-vintage patients are more common in recent practice [30, 31]. If similar secular trends occur among ICU patients receiving CKRT, dialysis vintage distribution among ESKD patients may have become more diverse over time. Because longer vintage was associated with a higher hazard of death within 90 days in our ESKD cohort, variation in dialysis vintage mix could influence study-level mortality patterns when ESKD is treated as a single category. This interpretation remains hypothesis generating and should be evaluated directly in time-stratified cohorts with explicit reporting of dialysis vintage.

Several limitations should be considered. First, the observational design precludes causal inference. Our analyses address associations rather than effects of modifiable interventions. Confirmation will require multicenter observational studies using methods to strengthen causal inference.

Second, this was a single-center study, and generalizability may be limited. CKRT practices differ across regions, including anticoagulation strategies. In our cohort, nafamostat mesylate was used in approximately 90% of patients. Nafamostat is commonly used for CKRT anticoagulation, particularly in Japan and South Korea [32]. Therefore, our findings should be interpreted cautiously in settings where other anticoagulants are routinely used and CKRT delivery characteristics may differ.

Third, ascertainment of death within 90 days was based on the electronic medical record at our hospital. Deaths after discharge or at other institutions may not have been completely captured. Patients without documented death and with available follow-up ending before 90 days were right censored at the last documented follow-up time. This may have introduced bias if censoring was informative. The proportion censored before 90 days differed across groups and was highest in the non-oliguric AKI group, which should be considered when interpreting the survival estimates.

Fourth, the sample size, particularly in the ESKD group (*n* = 66), was relatively small. As a result, our estimates may be imprecise, and the study may have been underpowered to detect small-to-moderate differences in the hazard of death within 90 days between groups. ESKD-restricted analyses, including the association with dialysis vintage, should therefore be interpreted with appropriate caution.

Fifth, pre-CKRT urine output was assessed from available clinical records within 24 h before CKRT initiation. Although urine output was standardized by body weight and assessment window, the duration and setting of assessment varied across patients. The restricted analysis limited to AKI patients with an assessment window of at least 6 h produced less precise estimates. Therefore, misclassification of oliguric and non-oliguric AKI remains possible, particularly among patients with short observation windows.

Sixth, several clinically relevant variables were unavailable or incompletely captured. Quantitative fluid balance, detailed vasoactive drug doses, echocardiographic cardiac function, and valvular disease at CKRT initiation were not available. We used chest radiography at CKRT initiation as a crude marker of cardiopulmonary congestion in a sensitivity analysis. However, chest radiography is non-specific and does not quantify fluid balance. Therefore, pre-CKRT urine output may reflect differences in volume status, hemodynamic management, or cardiac function in addition to kidney dysfunction, and residual confounding cannot be excluded.

## Conclusions

In ICU patients who started CKRT, pre-CKRT urine output in AKI and dialysis vintage in ESKD were associated with 90-day mortality. Non-oliguric AKI was associated with a lower adjusted hazard of death than ESKD, whereas oliguric AKI showed no clear difference. Within ESKD, longer dialysis vintage was associated with a higher hazard of death. These bedside variables require external validation in contemporary multicenter cohorts.

## Supplementary Information

Additional file 1.File 1. Predefined structured search and screening process. File 2. Search strategies for PubMedand Embase. File 3. Flow diagram of study identification and screening for the predefined structured search. File 4. Landmark Cox regression analyses for 90-day mortality after CKRT initiation. File 5. Baseline patient characteristics of the ESKD cohort stratified by dialysis vintage. File 6. CKRT settings at initiation in the ESKD cohort stratified by dialysis vintage. File 7. Kaplan–Meier curves for the exploratory four-group analysis. File 8. Cox regression analyses for the exploratory four-group analysis

## Data Availability

The datasets generated and/or analyzed during the current study are available from the corresponding author on reasonable request.
