# Supplementary material for: Prognostic associations of urine output in AKI and dialysis vintage in ESKD at continuous kidney replacement therapy initiation in ICU patients
Source: J Intensive Care. 2026 Aug 16;14:97. doi: 10.1186/s40560-026-00929-9 (PMC13543588; doi:10.1186/s40560-026-00929-9)
Supplement: Supplementary file 1 [file 40560_2026_929_MOESM1_ESM.docx]

**Additional file 1. Supplementary materials**

**Prognostic associations of urine output in AKI and dialysis vintage in ESKD at continuous kidney replacement therapy initiation in ICU patients**

Keisuke Okamoto, Naoki Ozu, Hidetada Fukushima, Masahiko Kawaguchi, Kazuhiko Tsuruya

**Contents:**

File 1. Predefined structured search and screening process.

File 2. Search strategies for PubMed (A) and Embase (B) (October 21, 2025).

File 3. Flow diagram of study identification and screening for the predefined structured search.

File 4. Landmark Cox regression analyses for 90-day mortality after CKRT initiation.

File 5. Baseline patient characteristics of the ESKD cohort stratified by dialysis vintage.

File 6. CKRT settings at initiation in the ESKD cohort stratified by dialysis vintage.

File 7. Kaplan–Meier curves for the exploratory four-group analysis.

File 8. Cox regression analyses for the exploratory four-group analysis.

**File 1. Predefined structured search and screening process.**

**Information sources and search date**

We conducted a predefined structured search in PubMed and Embase on October 21, 2025, from database inception to the search date.

**Search strategy**

We used controlled vocabulary terms and free-text keywords for acute kidney injury (AKI), end-stage kidney disease (ESKD), and intensive care unit (ICU) settings. We applied an English-language limit in both databases. In Embase, we excluded records indexed as conference abstracts. Stepwise electronic search strategies and yields are shown in Additional file 1: File 2.

**Record management**

We merged records retrieved from PubMed and Embase and removed duplicates. We then screened titles/abstracts and assessed full texts for eligibility (Additional file 1: File 3).

**Eligibility criteria**

We prespecified eligibility criteria to identify comparative studies relevant to the present cohort.

We included peer-reviewed full-text publications that:

1) enrolled adult ICU patients receiving kidney replacement therapy, including continuous modalities (CKRT/CRRT), and

2) reported original comparative outcome analyses between AKI and ESKD groups (including publications formatted as Letters when they contained extractable original comparative analyses).

We excluded publications without original comparative patient outcome analyses (e.g., narrative reviews, editorials/commentaries, and letters without extractable analyses), conference abstracts/proceedings, non-ICU settings, studies without an AKI–ESKD comparison, and reports without an extractable analysis restricted to kidney replacement therapy recipients.

**Title/abstract screening in Rayyan (exclusion reasons and operating rules)**

We performed title/abstract screening using Rayyan (rayyan.ai) with blinded decisions (reviewers were blinded to each other’s decisions). Two reviewers (K. Okamoto and N. Ozu) independently screened records and selected the first applicable exclusion reason according to a prespecified priority order (one reason per record). We used “Insufficient information / Full text needed” only when a decision was not possible from the title/abstract.

Exclusion reasons (priority order):

**1. Not original research (publication type)**: Review article, systematic review, meta-analysis, editorial, commentary, protocol, book chapter, case report, or letter without extractable original comparative analyses.

**2. Not CRRT/CKRT population (modality mismatch)**: Not CRRT/CKRT, or the CRRT/CKRT subgroup could not be separated.

**3. Wrong population (age/setting)**: Pediatric (<18 years), animal/in vitro, or setting not aligned with the protocol (e.g., non-ICU).

**4. No AKI vs ESKD comparison (wrong comparison/exposure)**: AKI-only or ESKD-only, or both appeared but without a direct AKI–ESKD comparison.

**5. Wrong outcome (non-patient outcomes)**: Non-clinical outcomes only (e.g., solute clearance, circuit performance) without patient-centered endpoints.

**6. Wrong study design (design mismatch)**: Otherwise aligned but design did not meet inclusion criteria (e.g., prognostic model development/validation only; methods/bench testing only).

**7. Insufficient information / Full text needed**: Key eligibility elements could not be confirmed from the title/abstract.

Disagreements were resolved by discussion.

**Full-text assessment and final inclusion**

We retrieved and assessed full texts for potentially eligible reports. A common full-text exclusion reason was the absence of an extractable analysis restricted to kidney replacement therapy recipients. The search identified 857 records (PubMed, n = 449; Embase, n = 408); 286 duplicates were removed; 571 records underwent title/abstract screening; 12 full texts were assessed; one report was excluded at full-text assessment because the ICU cohort was not restricted to patients receiving kidney replacement therapy and no extractable subgroup analysis was available; and 11 studies were retained (Additional file 1: File 3).

**Data extraction for the summary of prior studies**

From eligible studies, we extracted prespecified study characteristics and key mortality findings to populate the summary of prior comparative studies presented in the main text (Table 4).

**File 2. Search strategies for PubMed (A) and Embase (B)** **(October 21, 2025).**

(A) PubMed

| **No** | **Query** | **Results** |
| --- | --- | --- |
| #1 | "Acute Kidney Injury"[Mesh] OR "acute kidney injur*"[tiab] OR "acute kidney failure"[tiab] OR AKI[tiab] OR "acute renal failure"[tiab] OR "acute illness"[tiab] | 103016 |
| #2 | "Kidney Failure, Chronic"[Mesh] OR ESRD[tiab] OR ESKD[tiab] OR ESRF[tiab] OR "end-stage renal disease"[tiab] OR "end-stage renal failure"[tiab] | 133422 |
| #3 | "Intensive Care Units"[Mesh] OR ICU[tiab] OR "intensive care"[tiab] OR "critical care"[tiab] | 327031 |
| #4 | #1 AND #2 AND #3 | 470 |
| #5 | #4 AND English[la] | 449 |

(B) Embase

| **No** | **Query** | **Results** |
| --- | --- | --- |
| S1 | MJEMB.EXACT.EXPLODE("acute kidney failure") OR TI,AB("acute kidney injur*" OR "acute kidney failure" OR AKI OR "acute renal failure" OR "acute illness") | 143657 |
| S2 | MJEMB.EXACT.EXPLODE("chronic kidney failure") OR TI,AB(ESRD OR ESKD OR ESRF OR "end-stage renal disease" OR "end-stage renal failure") | 166105 |
| S3 | MJEMB.EXACT.EXPLODE("intensive care unit") OR TI,AB(ICU OR "intensive care" OR "critical care") | 481904 |
| S4 | S1 AND S2 AND S3 | 938 |
| S5 | DTYPE(conference abstract) | 5660598 |
| S6 | S4 NOT S5 | 423 |
| S7 | S6 AND LA(English) | 408 |

**File 3. Flow diagram of study identification and screening for the predefined structured search.**

**File 4. Landmark Cox regression analyses for 90-day mortality after CKRT initiation.**

| **Time interval** | **Group** | **Deaths / N (%)** | **Unadjusted HR (95% CI)** | **P** | **Adjusted HR (95% CI)** | **P** |
| --- | --- | --- | --- | --- | --- | --- |
| 0–7 days | ESKD | 16/66 (24.2) | Reference |  | Reference |  |
| 0–7 days | Oliguric AKI | 64/305 (21.0) | 0.85 (0.49–1.48) | 0.575 | 0.80 (0.45–1.42) | 0.441 |
| 0–7 days | Non-oliguric AKI | 23/189 (12.2) | 0.48 (0.25–0.90) | 0.023 | 0.56 (0.29–1.08) | 0.081 |
| 7–90 days | ESKD | 16/49 (32.7) | Reference |  | Reference |  |
| 7–90 days | Oliguric AKI | 79/237 (33.3) | 1.12 (0.65–1.91) | 0.684 | 0.93 (0.52–1.64) | 0.794 |
| 7–90 days | Non-oliguric AKI | 38/163 (23.3) | 0.74 (0.41–1.33) | 0.320 | 0.71 (0.39–1.31) | 0.277 |

Note: Deaths / N (%) indicates the number of deaths and the number of patients in each landmark interval before exclusion for missing covariates. Hazard ratios were estimated using Cox proportional hazards models with ESKD as the reference group. Unadjusted and adjusted Cox models were fitted using complete-case datasets for each landmark interval. The adjusted models were adjusted for age, sex, mean arterial pressure, body mass index, APACHE II score, diabetes mellitus, sepsis, mechanical ventilation use, vasopressor use, hemoglobin, serum albumin, and C-reactive protein. The 0–7-day analysis included patients at CKRT initiation, with follow-up administratively censored at 7 days. The 7–90-day analysis included patients who remained under follow-up beyond 7 days, with time reset at day 7. Oliguric AKI was defined as pre-CKRT urine output <0.5 mL/kg/h, and non-oliguric AKI was defined as pre-CKRT urine output ≥0.5 mL/kg/h. Abbreviations: AKI, acute kidney injury; APACHE, Acute Physiology and Chronic Health Evaluation; CI, confidence interval; CKRT, continuous kidney replacement therapy; ESKD, end-stage kidney disease; HR, hazard ratio.

**File 5. Baseline patient characteristics of the ESKD cohort stratified by dialysis vintage.**

| **Characteristics** | **Overall**  **(n = 66)** | **ESKD short-vintage**  **(n = 33)** | **ESKD long-vintage**  **(n = 33)** | **P** |
| --- | --- | --- | --- | --- |
| Age, years | 71 [64, 77] | 71 [62, 75] | 71 [66, 79] | 0.314 |
| Sex (Male), n (%) | 47 (71.2) | 25 (75.8) | 22 (66.7) | 0.415 |
| Body mass index, kg/m2 | 22.7 [19.0, 25.0] | 23.0 [19.9, 26.3] | 21.6 [17.6, 24.0] | 0.089 |
| Body weight, kg | 56.1 [47.7, 67.5] | 60.4 [55.0, 73.3] | 52.2 [44.7, 62.0] | 0.021 |
| Hypertension, n (%) | 41 (62.1) | 22 (66.7) | 19 (57.6) | 0.447 |
| Diabetes mellitus, n (%) | 33 (50.0) | 21 (63.6) | 12 (36.4) | 0.027 |
| Mean arterial pressure, mmHg | 76 [62, 82] | 77 [63, 90] | 75 [62, 79] | 0.373 |
| APACHE II score | 26 [22, 32] | 27 [23, 33] | 24 [21, 29] | 0.235 |
| Sepsis, n (%) | 30 (45.5) | 13 (39.4) | 17 (51.5) | 0.323 |
| Mechanical ventilation, n (%) | 44 (66.7) | 25 (75.8) | 19 (57.6) | 0.117 |
| Vasopressor use, n (%) | 46 (69.7) | 23 (69.7) | 23 (69.7) | 1.000 |
| ECMO use, n (%) | 1 (1.5) | 1 (3.0) | 0 (0.0) | 0.314 |
| IABP use, n (%) | 0 (0.0) | 0 (0.0) | 0 (0.0) | NA |
| Radiographic congestion at CKRT initiation, n (%) | 65 (98.5) | 33 (100) | 32 (97.0) | 0.314 |
| Hemoglobin, g/dL | 9.7 [8.2, 11.4] | 9.7 [8.4, 11.5] | 9.6 [8.2, 11.3] | 0.918 |
| Serum albumin, g/dL | 2.9 [2.4, 3.3] | 3.1 [2.5, 3.5] | 2.8 [2.4, 3.1] | 0.106 |
| Blood urea nitrogen, mg/dL | 52 [42, 75] | 58 [43, 81] | 50 [36, 72] | 0.180 |
| Serum creatinine, mg/dL | 7.2 [5.5, 8.8] | 7.3 [6.3, 9.4] | 6.5 [4.5, 7.9] | 0.187 |
| Serum potassium, mEq/L | 4.4 [4.0, 5.5] | 4.5 [4.0, 5.5] | 4.4 [4.0, 5.2] | 0.681 |
| C-reactive protein, mg/dL | 11.8 [2.8, 18.3] | 7.1 [2.6, 21.4] | 13.0 [5.0, 17.9] | 0.273 |
| pH on ABG | 7.36 [7.28, 7.41] | 7.36 [7.28, 7.41] | 7.36 [7.26, 7.40] | 0.995 |
| Bicarbonate on ABG, mEq/L | 21.0 [18.1, 23.3] | 21.0 [18.0, 22.9] | 21.1 [18.4, 23.4] | 0.720 |
| ICU admission diagnosis, n (%) |  |  |  | 0.426 |
| Sepsis/septic shock | 30 (45.5) | 13 (39.4) | 17 (51.5) |  |
| Cardiovascular | 3 (4.5) | 2 (6.1) | 1 (3.0) |  |
| Respiratory failure | 7 (10.6) | 6 (18.2) | 1 (3.0) |  |
| Cardiopulmonary arrest | 12 (18.2) | 5 (15.2) | 7 (21.2) |  |
| Postoperative/surgical | 5 (7.6) | 3 (9.1) | 2 (6.1) |  |
| Other shock | 8 (12.1) | 4 (12.1) | 4 (12.1) |  |
| Other | 1 (1.5) | 0 (0.0) | 1 (3.0) |  |
| Dialysis vintage, years | 5.6 [2.6, 11.3] | 2.6 [1.4, 4.3] | 11.6 [8.5, 17.7] | <0.001 |

Note: Data are presented as median [interquartile range] or n (%). ESKD short-vintage and long-vintage were defined by the median dialysis vintage (5.6 years) in the ESKD cohort. P values were calculated using the Mann–Whitney U test for continuous variables and Pearson’s χ² test for categorical variables. P values were not calculated for variables with no between-group variation. The main ICU admission diagnosis was classified according to the primary reason for ICU admission. Radiographic congestion at CKRT initiation was defined as the presence of any of the following on chest radiography: an enlarged cardiothoracic ratio suggestive of cardiomegaly, pulmonary congestion, or pleural effusion. Abbreviations: ABG, arterial blood gas; APACHE, Acute Physiology and Chronic Health Evaluation; CKRT, continuous kidney replacement therapy; ECMO, extracorporeal membrane oxygenation; ESKD, end-stage kidney disease; IABP, intra-aortic balloon pumping; ICU, intensive care unit.

**File 6. CKRT settings at initiation in the ESKD cohort stratified by dialysis vintage.**

| **Characteristics** | **Overall**  **(n = 66)** | **ESKD short-vintage**  **(n = 33)** | **ESKD long-vintage**  **(n = 33)** | **P** |
| --- | --- | --- | --- | --- |
| CKRT modality, n (%) |  |  |  | 0.314 |
| CVVHDF | 65 (98.5) | 33 (100) | 32 (97.0) |  |
| CVVHD | 1 (1.5) | 0 (0.0) | 1 (3.0) |  |
| Anticoagulation, n (%) |  |  |  | 0.352 |
| Nafamostat mesylate | 58 (87.9) | 28 (84.8) | 30 (90.9) |  |
| Heparin | 5 (7.6) | 2 (6.1) | 3 (9.1) |  |
| Argatroban hydrate | 1 (1.5) | 1 (3.0) | 0 (0.0) |  |
| None | 2 (3.0) | 2 (6.1) | 0 (0.0) |  |
| Hemofilter membrane type, n (%) |  |  |  | 0.564 |
| AN69ST | 15 (22.7) | 8 (24.2) | 7 (21.2) |  |
| PS | 50 (75.8) | 24 (72.7) | 26 (78.8) |  |
| CTA | 0 (0.0) | 0 (0.0) | 0 (0.0) |  |
| Unknown | 1 (1.5) | 1 (3.0) | 0 (0.0) |  |
| Initial blood flow rate, mL/min | 100 [100, 150] | 120 [100, 150] | 100 [80, 150] | 0.170 |
| Initial dialysate flow rate, mL/h | 300 [100, 500] | 300 [100, 500] | 300 [100, 500] | 0.973 |
| Initial replacement fluid flow rate, mL/h | 500 [300, 700] | 500 [300, 700] | 500 [300, 600] | 0.567 |
| Initial prescribed CKRT dose, mL/kg/h | 13.7 [11.9, 18.0] | 13.3 [11.4, 16.7] | 15.0 [12.7, 19.4] | 0.125 |

Note: Data are presented as median [interquartile range] or n (%). ESKD short-vintage and long-vintage were defined by the median dialysis vintage (5.6 years) in the ESKD cohort. P values were calculated using the Mann–Whitney U test for continuous variables and Pearson’s χ² test for categorical variables. CKRT settings, including modality and hemofilter membrane type, indicate initial settings at CKRT initiation. Unknown indicates that hemofilter membrane type was not documented in the CKRT prescription record. Initial prescribed CKRT dose was calculated as the sum of the initial dialysate flow rate and replacement fluid flow rate divided by body weight. Abbreviations: AN69ST, surface-treated AN69 membrane; CKRT, continuous kidney replacement therapy; CTA, cellulose triacetate; CVVHD, continuous venovenous hemodialysis; CVVHDF, continuous venovenous hemodiafiltration; ESKD, end-stage kidney disease; PS, polysulfone.

**File 7. Kaplan–Meier curves for the exploratory four-group analysis.**

Kaplan–Meier curves show cumulative survival probability within 90 days after CKRT initiation in the ESKD long-vintage, ESKD short-vintage, oliguric AKI, and non-oliguric AKI groups. Groups were compared using the log-rank test (P = 0.001). Numbers at risk are shown below the plot. ESKD short-vintage and long-vintage were defined by the median dialysis vintage (5.6 years) in the ESKD cohort. Oliguric AKI was defined as pre-CKRT urine output <0.5 mL/kg/h, and non-oliguric AKI was defined as pre-CKRT urine output ≥0.5 mL/kg/h. Abbreviations: AKI, acute kidney injury; CKRT, continuous kidney replacement therapy; ESKD, end-stage kidney disease.

**File 8. Cox regression analyses for the exploratory four-group analysis.**

| **Group** | **Deaths / N (%)** | **Unadjusted HR (95% CI)** | **P** | **Adjusted HR (95% CI)** | **P** |
| --- | --- | --- | --- | --- | --- |
| ESKD long-vintage | 20/33 (60.6) | Reference |  | Reference |  |
| ESKD short-vintage | 12/33 (36.4) | 0.49 (0.24–1.00) | 0.050 | 0.41 (0.20–0.85) | 0.017 |
| Oliguric AKI | 143/305 (46.9) | 0.71 (0.45–1.14) | 0.159 | 0.57 (0.35–0.92) | 0.022 |
| Non-oliguric AKI | 61/189 (32.3) | 0.44 (0.27–0.73) | 0.001 | 0.42 (0.25–0.70) | 0.001 |

Note: Hazard ratios were estimated using Cox proportional hazards models with ESKD long-vintage as the reference group. Deaths / N (%) indicates the number of deaths and patients before exclusion for missing covariates. The adjusted Cox model was fitted using complete-case analysis and was adjusted for age, sex, mean arterial pressure, body mass index, APACHE II score, diabetes mellitus, sepsis, mechanical ventilation use, vasopressor use, hemoglobin, serum albumin, and C-reactive protein. ESKD short-vintage and long-vintage were defined by the median dialysis vintage (5.6 years) in the ESKD cohort. Oliguric AKI was defined as pre-CKRT urine output <0.5 mL/kg/h, and non-oliguric AKI was defined as pre-CKRT urine output ≥0.5 mL/kg/h. This analysis was exploratory. Abbreviations: AKI, acute kidney injury; APACHE, Acute Physiology and Chronic Health Evaluation; CI, confidence interval; CKRT, continuous kidney replacement therapy; ESKD, end-stage kidney disease; HR, hazard ratio.
